# Using modelled relationships and satellite observations to attribute modelled aerosol biases over biomass burning regions

**DOI:** 10.1038/s41467-022-33680-4

**Published:** 2022-10-07

**Authors:** Qirui Zhong, Nick Schutgens, Guido R. van der Werf, Twan van Noije, Susanne E. Bauer, Kostas Tsigaridis, Tero Mielonen, Ramiro Checa-Garcia, David Neubauer, Zak Kipling, Alf Kirkevåg, Dirk J. L. Olivié, Harri Kokkola, Hitoshi Matsui, Paul Ginoux, Toshihiko Takemura, Philippe Le Sager, Samuel Rémy, Huisheng Bian, Mian Chin

**Affiliations:** 1 Department of Earth Sciences, Vrije Universiteit Amsterdam, Amsterdam, The Netherlands; 2 Royal Netherlands Meteorological Institute, De Bilt, The Netherlands; 3 NASA Goddard Institute for Space Studies, New York City, NY USA; 4 Center for Climate Systems Research, Columbia University, New York City, NY USA; 5 Finnish Meteorological Institute, Kuopio, Finland; 6 Laboratoire des Sciences du Climat et de l’Environnement, IPSL, Gif-sur-Yvette, France; 7 Institute of Meteorology and Climatology, University of Natural Resources and Life Sciences, Vienna, Austria; 8 Institute for Atmospheric and Climate Science, ETH Zurich, Zurich, Switzerland; 9 European Centre for Medium-Range Weather Forecasts, Reading, UK; 10 Norwegian Meteorological Institute, Oslo, Norway; 11 Graduate School of Environmental Studies, Nagoya University, Nagoya, Japan; 12 NOAA, Geophysical Fluid Dynamics Laboratory, Princeton, NJ USA; 13 Research Institute for Applied Mechanics, Kyushu University, Fukuoka, Japan; 14 HYGEOS, Lille, France; 15 University of Maryland, Baltimore County (UMBC), Baltimore, MD USA; 16 NASA Goddard Space Flight Center, Greenbelt, MD USA

**Keywords:** Atmospheric science, Climate and Earth system modelling, Environmental sciences

## Abstract

Biomass burning (BB) is a major source of aerosols that remain the most uncertain components of the global radiative forcing. Current global models have great difficulty matching observed aerosol optical depth (AOD) over BB regions. A common solution to address modelled AOD biases is scaling BB emissions. Using the relationship from an ensemble of aerosol models and satellite observations, we show that the bias in aerosol modelling results primarily from incorrect lifetimes and underestimated mass extinction coefficients. In turn, these biases seem to be related to incorrect precipitation and underestimated particle sizes. We further show that boosting BB emissions to correct AOD biases over the source region causes an overestimation of AOD in the outflow from Africa by 48%, leading to a double warming effect compared with when biases are simultaneously addressed for both aforementioned factors. Such deviations are particularly concerning in a warming future with increasing emissions from fires.

## Abstract (web summary)

Error attribution based on modelled relationships and satellite observations suggests that errors in global models are more important and require more concerns than emission errors in creating the overall uncertainties for biomass burning aerosols.

## Introduction

Biomass burning (BB) injects large amounts of aerosols into the atmosphere, which significantly affects the Earth’s climate, human health, and ecosystems^1–3^. Recent evidence points towards an increasing trend in fires over several forest fire-dominated BB regions, such as Siberia, North America, and Australia, resulting in substantially high BB aerosol (BBA) emissions^4–7^. With human-induced climate change in the future, such an increasing trend is likely to continue in many forested regions, suggesting even stronger BBA impacts, which could lead to BB becoming a more important source of aerosol than those with a direct anthropogenic origin^8,9^.

However, our current understanding of the impacts of BBA remains weak. The net radiative effect of BBA is highly uncertain ranging from strong warming to substantial cooling effects^10–13^, resulting mostly from the inaccurately represented distribution and properties of clouds and BBA^14,15^. The latter uncertainty is often linked with BBA emissions, as their estimates differ by a factor of 3.8 (for organic aerosols) on a global scale with even larger mismatches on regional scales^16^. Thus, global aerosol models driven by these emissions show large diversities in simulating aerosol optical depth (AOD) for BBAs, but with underestimations by a factor of 2−5 commonly observed^17–20^. Consequently, BBA emissions are often inflated to bring modelled AOD more in line with observations^14,17–19,21^, while other model aspects are assumed to be reasonable. This correction may mask other potential parametric and/or structural errors in models, including (but not limited to) simplified assumptions on the aerosol mixing state^22,23^, missing ageing and coating mechanisms^24–26^, incorrect particle size distribution^27^, inaccurate aerosol hygroscopicity^19,28^, and misrepresented removal processes^29^. However, the relative importance of these errors for total BBA errors is still unclear. A quantitative assessment of BBA errors in terms of multiple aspects would greatly contribute to better modelling of aerosols which remain the most uncertain climate forcers^30^.

**Fig. 1:**
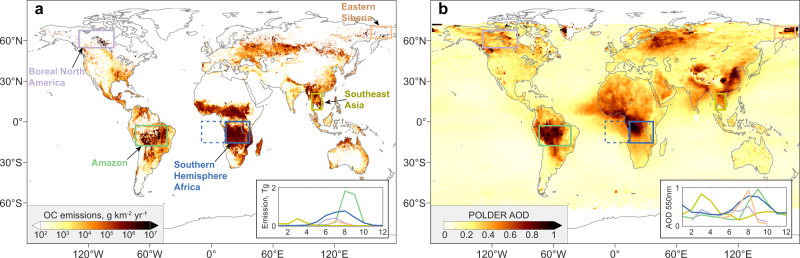
Geophysical distribution of biomass burning emissions and aerosol optical depth (AOD) in 2010. **a** Annual organic carbon (OC) emissions based on GFED (https://www.globalfiredata.org)^35^. **b** Annual mean AOD at 550 nm from POLDER satellite observation (https://www.grasp-open.com)^43^. The boxes indicate the spatial coverage of the five key biomass burning regions focused on here. The dashed box shows the African outflow region of focus in this study. The embedded diagrams show the corresponding monthly total emissions (**a**) and mean AOD (**b**). The line colours in the embedded diagrams correspond to the boundaries in the maps.

Here, we investigate AOD errors over five key BB regions (see Fig. 1) in the context of a well-known equation:

1 $${AOD}=E\times \tau \times {MEC}$$

where *E* is total emissions, *τ* is the lifetime (defined as burden/emissions), and *MEC* is the mass extinction coefficient (defined as AOD/burden), and we solve the equation on a regional scale averaged over the fire seasons. In this study, we adopted the following procedure: 1) we established linear regressions for lifetime over precipitation and Ångström Exponent (AE, an indicator of ambient particle size) and between MEC and AE in an ensemble of models from the Aerosol Comparisons between Observation and Models project (AeroCom, see Supplementary Table 1); 2) we estimated the constrained lifetime and MEC from these regressions and satellite observations of precipitation and AE; 3) constrained emissions were then estimated from Eq. (1) using satellite observations of AOD; and 4) finally, AOD errors in individual models were attributed to emission, lifetime and MEC contributions. This method allowed for an integrated assessment of model AOD errors based on three interpretable components that are constrained using observational datasets. Such a quantitative attribution of AOD errors due to multiple sources has, to our knowledge, not been conducted previously. The resulting information can be immediately used to improve model performance and has several key implications detailed below. Notably, our constraining analysis was conducted on a seasonal and regional scale. The constrained results cannot be directly applied to a smaller scale within the regions. Similarly, evaluations/interpretations of individual fires are beyond the scope of this study.

## Results

### Estimating the constrained lifetime and MEC

**Fig. 2:**
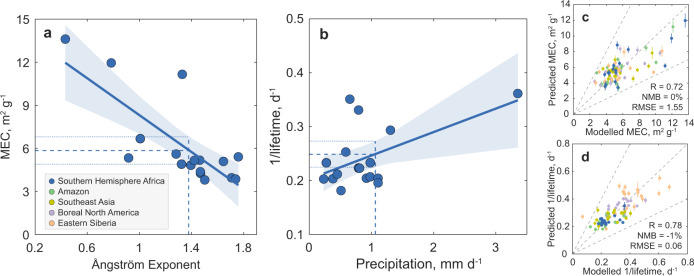
Linear regressions developed over AeroCom models to estimate the constrained mass extinction coefficient (MEC) and lifetime. **a** The linear regression for MEC over Ångström Exponent. **b** The linear regression for lifetime over precipitation. Note that the real lifetime regression uses both precipitation and the Ångström Exponent as predictors given the regression improvement (Supplementary Fig. 2). In **a** and **b**, the regressions are shown for Southern Hemisphere Africa as an example with each dot indicating the regional and fire-season mean values in one model. The solid lines indicate the linear regressions together with the 95% confidence intervals (shaded areas). The regional mean observations of POLDER Ångström Exponent^43^ (**a**) and GPCP precipitation (**b**) are shown as vertical dashed lines, and the corresponding horizontal dashed lines show the constrained MEC (**a**) and lifetime (**b**) and 95% confidence intervals (dotted lines). **c**, **d** Comparisons between the regression predictions and the original model data over the five regions with error bars showing 50% prediction uncertainties. The colours of the dots indicate data in the five regions. The metrics for all the regions show the correlation coefficient (R), normalized mean bias (NMB), and root mean square error (RMSE). Details of the regressions and validations can be found in the Methods.

The key to understanding AOD model errors due to the three sources (emissions, lifetime, and MEC) is to estimate their observation-constrained values. As a first step towards constraining MEC, we found that the AeroCom models suggested a rather linear relationship between modelled MEC and AE (see Fig. 2a as an example for Southern Hemisphere Africa and Supplementary Fig. 1 for other regions), as both are associated with particle size^31^. Similarly, we built linear regressions between the modelled lifetime and precipitation due to the dominant impact of precipitation on wet removal, which explains most of the variations in lifetime^29^. The regression was further improved by including AE as an additional predictor (see Supplementary Fig. 2) since aerosol removal is relevant to particle size^32^. The updated lifetime and MEC regressions were validated using original model data, suggesting good agreement (see Fig. 2c, d). By comparing the two predictors with the observations, we found that the modelled precipitation deviated substantially from the observations by a factor of 0.2−3.1, whereas the modelled AEs were overestimated by more than half the models.

Then, we employed the regional observations of precipitation and AE (see Methods) to the above linear relationships to constrain lifetime and MEC for each region individually (Fig. 2). Unlike the modelled lifetime that straddles the constrained values, the MEC in most models was underestimated (see Table 1). This finding was further confirmed by comparisons of the modelled MEC with data collected from flight campaigns (Supplementary Fig. 3a), where the observations suggested a larger MEC than most models. Moreover, evaluation of particle radius in an individual model also indicated that the modelled particle size was underestimated (see Supplementary Fig. 3b for ECHAM-HAM model, also found in different models by, e.g., Chin et al.^27^ and Brown et al.^22^). Notably, the comparisons of modelled particle size and MEC with observations were affected by sampling errors due to the mismatch in time and space (see Supplementary Tables 2, 3). However, underestimation of MEC for BBA was reported in previous studies (e.g., ref. 28,33) and was also supported by data presented in Doherty et al. ^13^.

**Table 1:** The constrained results from the present study compared with AeroCom models and observations

		Amazon	Southern Hemisphere Africa	Southeast Asia	Boreal North America	Eastern Siberia
Precipitation (mm d^−1^)	GPCP^a^	1.9	1.1	0.8	1.8	0.6
	Models	2.1 [1.6, 2.3]	0.8 [0.5, 1]	0.9 [0.5, 1.7]	2.3 [2, 2.5]	1 [0.7, 1]
AE	This study^b^	1.4 [1.3, 1.4]	1.4 [1.3, 1.4]	1.2 [1.2, 1.3]	1.3 [1.2, 1.4]	1.1 [1, 1.2]
	Models	1.4 [1.3, 1.7]	1.4 [1.2, 1.5]	1.2 [1.1, 1.5]	1.5 [1.1, 1.8]	1.5 [1.4, 1.9]
AOD	This study^b^	0.4 [0.38, 0.42]	0.56 [0.53, 0.59]	0.88 [0.84, 0.93]	0.16 [0.15, 0.18]	0.21 [0.19, 0.23]
	Models	0.32 [0.29, 0.43]	0.34 [0.27, 0.44]	0.45 [0.41, 0.56]	0.13 [0.09, 0.2]	0.21 [0.16, 0.32]
Total emission^c^ (10^−11 ^kg m^−2^ s^−1^)	This study	18.2 [16.4, 20.3]	27.9 [24.4, 32.5]	47.6 [42.4, 53.7]	10.3 [9.1, 11.5]	8.3 [7, 9.8]
	Models	17.3 [14.1, 23]	12.7 [12.5, 23.7]	24.9 [17.7, 38.1]	7.8 [6.5, 11.5]	16 [13.4, 28.1]
Lifetime (d)	This study	4.3 [4.1, 4.5]	4.0 [3.8, 4.1]	3.9 [3.6, 4.1]	3 [2.9, 3.1]	4.4 [4, 4.7]
	Models	4.5 [3.8, 5.3]	4.5 [3.8, 4.9]	3.9 [2.9, 5.1]	2.8 [2.6, 3.3]	2.6 [2.1, 2.9]
MEC (m^2^ g^−1^)	This study	5.9 [5.3, 6.5]	5.9 [5.2, 6.6]	5.6 [5.2, 5.9]	6 [5.6, 6.4]	6.8 [6.3, 7.4]
	Models	5 [4.1, 5.8]	5.2 [4.3, 5.9]	5 [4.4, 6.1]	5.6 [4.6, 6.9]	5.1 [3.9, 5.8]
	Observation^d^	7.5 [6.2, 10.7]	9.7 [6.5, 15.3]		5.4 [3.9, 7.9]	

Data in brackets show the interquartile ranges.

^a^Precipitation observation is from Global Precipitation Climatology Project version 2.3 (GPCP).

^b^Values denote the regional averages estimated based on POLDER data.

^c^Total emissions include both BBA and background emissions.

^d^Observations are from flight campaigns in Supplementary Table 2.

### Constraining BBA emissions

**Fig. 3:**
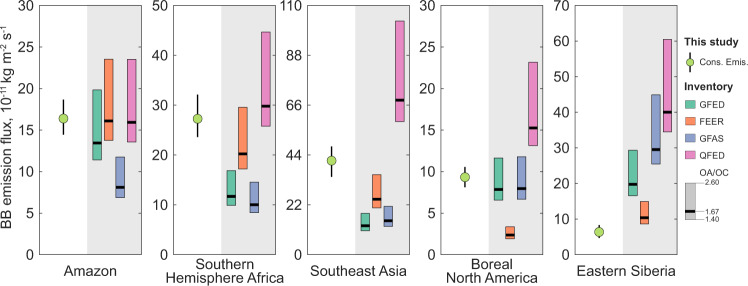
Comparisons of biomass burning emission flux between this study and four emission inventories. The constrained BB emissions (Cons. Emis.) are shown with interquartile ranges (error bars) resulting from the eight uncertainty factors (see Supplementary Fig. 4). Total BB emissions consist of OA, BC, and SO_2_. The OA emissions in the four inventories are converted from OC emissions with a ratio ranging from 1.4 to 2.6 as used in the AeroCom models (shown as the range of bars). The 1.67 OA/OC ratio (thick black lines) from a recent field measurement is used for direct comparison^37^. The four inventories are Global Fire Emission Database version 4.1 s (GFED)^35^, Fire Energetics and Emission Research version 1.2 (FEER)^34^, Global Fire Assimilation System version 1.2 (GFAS)^18^, and Quick Fire Emissions Dataset version 2.5 (QFED)^36^.

After obtaining the constrained lifetime and MEC, we used regional AOD observations to estimate constrained total emissions (see Table 1). These total emissions included secondary organic aerosol (SOA) formation given the shorter formation time scale compared with the time scale of our analysis (fire seasons). By excluding “background” emissions (i.e., dust, sea salt, biogenic, and anthropogenic aerosols) that accounted for less than 10% of total aerosol emissions (see Methods), we compared the constrained BBA emissions with four emission inventories (Fig. 3), including one bottom-up dataset based on burned area and modelled fuel load (Global Fire Emission Database version 4.1 s [GFED]) and three top-down datasets relying on fire radiative power (i.e., Quick Fire Emissions Dataset version 2.5 [QFED]; Fire Energetics and Emission Research version 1.2 [FEER]; Global Fire Assimilation System version 1.2 [GFAS])^18,34–36^. Note that the BBA emissions were considered for species of black carbon (BC), organic aerosol (OA), and sulfur dioxide (SO_2_), with dominance from the former two carbonaceous components. To compare these inventories with our results, we converted the organic carbon (OC) emissions from the four inventories to OA emissions by adopting the OA/OC ratio from the AeroCom models (1.40–2.60) together with a recent field measurement (1.67) for freshly emitted BBA^37^. The total BBA emissions from the four inventories varied substantially by a factor of 3–10 for the five regions. Our constrained emissions showed a smaller spread with fully considered uncertainties resulting mainly from satellite retrieval errors (see Methods and Supplementary Fig. 4). As seen, GFED, FEER, and QFED agreed well with our constrained BBA emissions over the Amazon, suggesting that modelled AOD errors when using these inventories were probably determined by the errors in lifetime and MEC (similar for GFED and GFAS in Boreal North America). In Southern Hemisphere Africa and Southeast Asia, three of the inventories (GFED, FEER and GFAS) were significantly lower than our estimates, implying missing emissions^16,38,39^. In contrast, QFED was much higher and likely overestimated emissions over Africa and Southeast Asia. In Eastern Siberia, inventories tended to be much higher except for FEER. This analysis also suggested that the models exhibited different errors in each region, which will require regionwise strategies to fix the bias in the current emission inventories. With the OA/OC of 1.67 used^37^, the average deviations for the five fire regions of the four inventories relative to our estimations were −36%, −28%, −31%, and +69% for GFED, FEER, GFAS, and QFED, respectively. Obviously, such deviations can be largely altered with different choices of OA/OC. Further investigations on this important parameter could reduce emission uncertainties. Nevertheless, there are still significant differences between some inventories and our constrained emissions after considering the uncertainty in the OA/OC ratio, suggesting biases in the inventories.

### Attributing AOD errors to emissions, lifetime and MEC

**Fig. 4:**
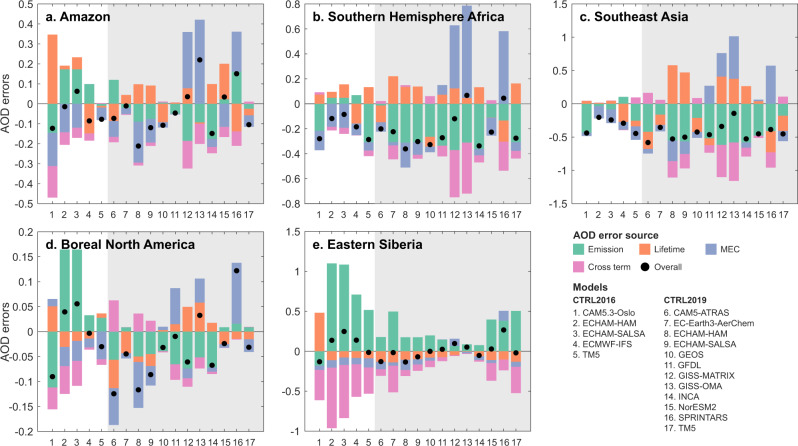
Aerosol optical depth (AOD) error attribution for AeroCom models. Total mean AOD errors during the BB seasons (black dots) are attributed to the errors in total emission, lifetime, and MEC over the five BB region. The cross terms show the interactions among the three factors. The uncertainties of the errors attributable to the three factors can be found in Supplementary Fig. 5 (see Methods).

Model errors in AOD can now be attributed by comparing a model’s emissions, lifetime, and MEC with the constrained values (see Methods). Figure 4 shows that a significant balancing of errors existed in almost all models. We found a substantial bias induced by emissions, mostly underestimation except for the Amazon and Eastern Siberia regions, as mentioned previously. The differences in the emission-related bias were associated with the choices of emission datasets and assumptions on OA/OC ratios in the models (see Supplementary Table 1). However, AOD errors cannot be fully explained without considering errors related to lifetime and MEC, which either compensated for or exacerbated errors caused by emissions. In fact, AOD errors were less contributed by the emission errors (38 ± 19%) than the total errors induced by MEC (27 ± 19%) and lifetime (22 ± 15%) errors for the whole ensemble of models. The cross effects between the three factors were nonnegligible but less important (13 ± 9%) for the whole ensemble, although individual models contained large cross-term errors. Despite the uncertainties shown in Supplementary Fig. 5, we observed negative errors due to MEC in most models, suggesting a common issue of modelled particle sizes that are too small. Although some models showed near perfect agreement with AOD observations (e.g., ECHAM-HAM model from CTRL2016 over Amazon), it does not necessarily guarantee the reliability of all three modelled aspects, as the overall agreement could result from compensation of substantial individual deviations. Consequently, estimating BBA emissions based merely on AOD observations (as is done for QFED^36^) could result in strong uncertainties related to the modelled lifetime and MEC. Furthermore, the compensation among the errors from the three aspects varied substantially per model and region. This finding suggested that different strategies should be taken when handling these errors instead of focusing on emissions only.

### Estimating African outflow AOD in AeroCom models

**Fig. 5:**
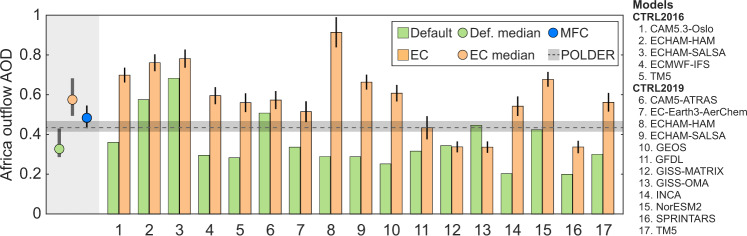
Estimated aerosol optical depth (AOD) over the outflow region of Southern Hemisphere Africa for AeroCom models. The Default results refer to the original model output. Two corrections (EC and MFC) are adopted over the source region (Southern Hemisphere Africa) only, and their results are based on our multivariate linear regression for AeroCom models. For the EC case, only emissions are rescaled according to the AOD errors in the source region. For the MFC case, emission, lifetime, and MEC are corrected to our constrained values. The results for default, EC, and MFC cases are shown in green, orange, and blue, respectively. The spread of the model ensemble for default and EC cases are shown as dots (median) and the interquartile ranges (thick grey error bars) on the left. The thin error bars of EC case for individual models (1-17) and the MFC case show the 50% confidence intervals which considered the uncertainties in the input data (i.e., the rescaled emission in the EC case and the constrained values in the MFC case) and the meta-model regression (see Methods). Note that the MFC prediction is shown as a single dot (with error bar) since it is obtained from a single prediction by using the constrained emission, lifetime, and MEC (see Methods). For validation, the POLDER observation^43^ is presented as the median (black dashed line) and interquartile (grey shaded area) obtained from the construction of regional AOD (see Methods).

Better constraining BBA has profound implications not only on the source regions but also on the outflow areas since BB plumes are usually transported over a long distance. Based on the AeroCom model data, we constructed a multivariate linear model to predict the AOD over the African outflow region (given that Africa has the highest emissions of all continents) in response to the emissions, lifetime, and MEC in the source region (see Methods). Then, we investigated the impact on outflow AOD of two different methods of correcting AOD errors in the source region: 1) Using the default model configuration but increasing BBA emissions in the source region to match the observed AOD, as was done in previous studies (emission-based correction, or EC, see Methods); or 2) emissions, lifetime, and MEC were simultaneously corrected to the constrained values (multifactor correction, or MFC). The estimated outflow AODs for the two corrections are shown in Fig. 5 for all the AeroCom models, indicating general overestimations in the EC case because too much BBA was injected into the atmosphere. In contrast, the MFC case agreed better with the observation, which supported the validity of our error attribution methodology. This finding highlighted that addressing lifetime and MEC errors would greatly reduce the overall BBA uncertainties in both source and vast outflow regions.

### Correcting global model (ECHAM-HAM) simulations

**Fig. 6:**
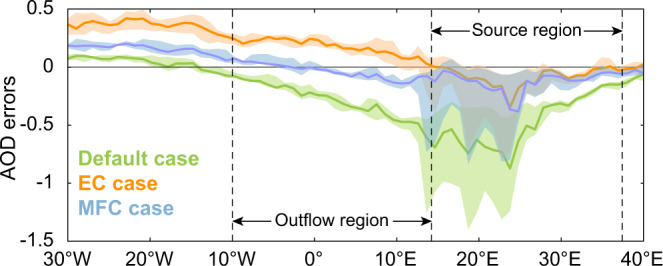
Modelled aerosol optical depth (AOD) errors in the source and outflow region of Southern Hemisphere Africa by ECHAM-HAM model. All the simulations are collocated and compared with POLDER satellite AOD data^43^ during the fire season. Simulations are performed in three cases: 1) default case, 2) EC case, where we only increase the emissions in the source region to match AOD observations; and 3) MFC case, where we modify the emission, precipitation (for correcting lifetime), and particle size (for correcting MEC). The results are shown as the median (solid lines) and interquartile (shaded areas) of the seasonal AOD errors for all grid cells within the latitudinal range shown in Supplementary Fig. 6. Details about the three cases can be found in Methods.

Furthermore, we carried out a model simulation with the ECHAM-HAM model which had its wet deposition efficiency and emitted particle size modified to better agree with our constrained lifetime and MEC (MFC case, see Methods). This model performed much better over the African source region compared with the default case, reducing the regional AOD errors from −62% to −10% (Fig. 6). In addition, it also corrected the AOD underestimation in the outflow region, with much better performance than a simulation in which only emissions were adjusted, which led to a large overestimation (EC case, see Fig. 6 and Supplementary Fig. 6). The EC simulation resulted in many more particles that can serve as cloud condensation nuclei, which may modify the formation of clouds and affect precipitation^40^. Note that in these two simulations, the existing north-south gradient in the AOD errors was not entirely corrected (see Supplementary Fig. 6). Studies focusing on local instead of regional dynamics are needed to better understand this remaining uncertainty.

The all-sky instantaneous direct radiative effect (IDRE) (Supplementary Fig. 7) was also affected by how we corrected the model. As with AOD, we observed a notable difference over the southern Africa outflow region, where the EC simulation showed a much stronger warming effect (20.5 ± 11.5 W/m^2^) than the MFC case (9.5 ± 5.1 W/m^2^). We further compared the IDRE with previous studies based on flight campaigns and satellite observations (Supplementary Table 4). The reported IDRE differs substantially and is partly driven by different assumptions on cloud cover and properties. Despite large uncertainties, the reported IDREs were generally lower than the EC prediction, suggesting a possible overestimation of the warming effects in EC. The strong warming in the EC simulation was associated with superabundant absorbing aerosols (e.g., BC, see Supplementary Fig. 8). The increases in the absorbing aerosols would further modify the stratocumulus cloud properties and atmospheric circulation, introducing extra uncertainties in the radiative budget^41,42^. Note that the MFC simulation yielded better agreement with independent observations of absorbing aerosol than the EC simulation, even though such observations were not used in identifying or correcting model errors (see Supplementary Fig. 8).

## Discussion

By evaluating an ensemble of global models from the AeroCom project against satellite observations, we were able to attribute modelled AOD errors for BBAs over source regions to incorrect emissions, lifetimes, or MECs. This error attribution was further validated with a simple estimate of the outflow AOD for the African BBA, flight campaign in situ observations, and detailed model simulations. Our results show that errors in lifetime and MEC contribute significantly to the overall error in modelled AOD, which previously have been improperly attributed to errors in emissions. Moreover, AOD errors usually result from multiple error sources that frequently balance each other. Consequently, scaling emissions to obtain a better agreement with observations leads to errors in outflow regions. In turn, this has implications for the BB aerosol forcing. As an example, our model simulations suggested that only scaling emissions to match AOD could introduce substantial biases in the magnitude of radiative forcing in the outflow regions. Such biases are likely to be exacerbated in the future, as most climate models project more prevailing fires in key BB regions^8,9^. For such foreseeable fires, our method would benefit understanding and addressing potential BBA errors in global aerosol models. With proper extensions, this method could be further applied to other aerosol sources, regions, and possibly even global studies. Accounting for model errors using this method would contribute to narrowing down the overall aerosol uncertainties in atmospheric and climate research with relatively smaller computational costs than perturbed parameter ensembles or data assimilation experiments.

Our work presents an attempt to quantitatively distinguish the role of emissions versus model parameterizations (with a focus on lifetime and MEC) in creating AOD errors of BBA. Although there have been studies discussing the existence of errors in both models and emissions, only qualitative conclusions could be reached, and the contributions of these error sources to the overall AOD error are still missing. Our results highlight the priority of addressing model biases to properly represent BBAs, which also has the potential to improve the representation of other (anthropogenic) aerosol sources. This work is based on a large model ensemble, which can avoid systematic biases in a specific model, although uncertainties exist. In particular, we find that the satellite retrieval error of AE plays a significant role in the overall uncertainties of our work. The uncertainties due to satellite retrieval errors can be reduced by using the AERONET dataset, which cannot significantly reduce the overall uncertainties due to the large sampling biases with poor data coverage (see Methods and Supplementary Fig. 9). Therefore, future satellite products with improved data quality (low retrieval errors) and increased data coverage would greatly reduce the uncertainties of our work.

## Methods

### Biomass burning regions

The study focused on aerosols over five key BB regions: the Amazon, Southern Hemisphere Africa, Southeast Asia, Boreal North America, and Eastern Siberia. The fire season in 2010 was defined for each region based on recorded BBA emissions and satellite-observed AOD peaks. The spatial distribution and monthly evolution of BBA emissions and AOD over the five BB regions are mapped in Fig. 1. According to the GFED database, the major burned biome types that contributed significantly to the global BB aerosol budget were included in these five BB regions (i.e., tropical forest, savanna, grassland, and boreal forest). In total, emissions from these five regions accounted for more than half of the global total BBA emissions for BC and OC^35^. Based on BBA emissions and AOD observations, we defined the BB seasons in 2010 as follows: July to October for Amazon, June to September for Southern Hemisphere Africa, March for Southeast Asia, June to August for Boreal North America, and July for Eastern Siberia.

### AeroCom models and observation data

We used modelled AOD data at 550 nm from two control experiments (CTRL2016, CTRL2019) in phase III of the Aerosol Comparisons between Observations and Models project (AeroCom). AeroCom is an open international initiative aiming at a better understanding of global aerosols and their impact. The two phase III control experiments represent the state-of-the-art aerosol modelling for 2010. A total of 17 models were involved, as detailed in Supplementary Table 1. In addition to AOD data, data on emissions, aerosol burdens, precipitation, and Ångström Exponent (AE, calculated from AOD at 550 nm and 440 nm) were also used.

Observations of AOD and AE in 2010 were taken from the POLarization and Directionality of the Earth’s Reflectances (POLDER) sensor on the PARASOL platform using the Generalized Retrieval of Atmosphere and Surface Properties algorithm^43^. The POLDER data were validated previously on the global scale and have shown better performance than other satellite datasets^44,45^. We used precipitation from the Global Precipitation Climatology Project (GPCP) version 2.3 as observations. GPCP combines satellite estimates and ground measurements to estimate precipitation globally and has been used to evaluate global climate models before^46,47^. BBA particle size and MEC from field measurements and flight campaigns were also collected to further verify the modelled aerosol properties (see Supplementary Tables 2, 3).

The modelled AOD was first regridded into 1° × 1° grid cells and compared against POLDER observations using 3-hour average data for CTRL2016 models and daily data for most CTRL2019 models. The normalized mean bias for the five fire regions is listed in Supplementary Table 1, showing a diverse, generally negative bias.

The constraining procedures in this study required regional average observations. We estimated regional AOD (and AE) based on the combination of models and raw POLDER data using the modelled linear relationships between regional average AOD and sampled AOD (see Supplementary Method 1 and Supplementary Fig. 10). Note that models with monthly output were not used for reconstructing the regional AOD (and AE) considering the substantial sampling issues^48,49^.

### Constraining emission, lifetime, and MEC

We used Eq. (1) to distinguish the three aspects of the modelled AOD errors. The regional and seasonal averages of emissions, aerosol burden, and AOD were used to calculate lifetime (= burden/emission) and MEC (= AOD/burden) to present a fire-season average. Emissions, burden, and AOD were totalled for all aerosol species in each model, including OA, BC, sulfate (including SO_2_ in emissions), sea salt, and dust. Given the short time scale of SOA formation compared to the fire seasons analysed here, we treated SOA formation as part of the total emissions. We additionally assumed that the ageing of SOA did not modify the total aerosol mass and properties significantly on a seasonal scale, since the overall ageing mechanism remained highly uncertain and poorly understood^50–52^. Our meta-model for Africa outflow AOD could successfully reproduce POLDER observations (see Fig. 5), suggesting that these assumptions were acceptable.

Equation (1) has been used to intercompare models^53^, and individual factors have also been studied from an observational point^28^. To constrain lifetime and MEC, we built linear regressions within the AeroCom model ensemble for each BB region (see Fig. 2 and Supplementary Fig. 1) with the following forms:

2 $$1/\tau={a}_{1}{Pr}+{a}_{2}{AE}+{a}_{3};{MEC}={b}_{1}{AE}+{b}_{2}$$

where *Pr* denotes the total precipitation strength. For lifetime, we also found a weak correlation between lifetime and plume heights in addition to the above two predictors, but it was inconsequential for our analysis. Similarly, we also found that MEC in specific models was related to relative humidity and/or single-scattering albedo. These relationships were relatively weak for the whole model ensemble and had little effect on our results. By employing the observations of precipitation and AE to these regressions, we then constrained lifetime and MEC. Compared with modelled precipitation and AE, which exhibit large diversities among models, the observed precipitation and AE and the constrained lifetime and MEC are within the model spread, lending further credibility to the estimates. Using the regional AOD, we finally calculated the constrained total emissions based on Eq. (1). Constrained BBA emissions were further estimated by subtracting separate estimates of background emissions, including anthropogenic aerosols, biogenic SOA, dust, and sea salt (see Supplementary Table 5). In total, these background emissions contributed less than 10% of the constrained total aerosol emissions. Note that the constrained BBA emissions also included SOA production from the BB source. All the constrained values are detailed in Table 1 in comparison with the model and observation data.

To verify the robustness of our approach for constraining these three components, we tested the methodology by removing one model and assuming it to be the truth. The corresponding modelled emission, lifetime, and MEC could then be compared against the values constrained from the remaining models, as shown in Fig. 2c, d and Supplementary Fig. 11. These comparisons suggested good agreement between the originally modelled and predicted values, demonstrating the reliability of this approach. In addition, we also tested the robustness of the constrained estimates by excluding one of the models from the ensemble or removing two models with the highest/lowest precipitation (or AE). As shown in Supplementary Fig. 12, this will mostly give very similar estimates of constrained values as the original analysis based on all models, suggesting that our results were insensitive to extreme values from specific models. This further demonstrated the robustness of our predictions.

### Error attribution

Based on Eq. (1), we calculated the modelled AOD errors due to one component (total emission, lifetime, and MEC) by multiplying the error in that component with the constrained value of the other two components. Equation (3) gives an example of emission-induced errors (*ε*_*E*_).

3 $${\varepsilon }_{E}=\triangle E\times {\tau }_{0}\times {{MEC}}_{0}$$

We also introduced an error cross term that contained the difference between the full model AOD error and the sum of errors from all three components. This result indicated the error interactions among the three components.

### Uncertainty estimation

We utilized a Monte-Carlo approach to quantify the uncertainties of our analysis resulting from 8 uncertain parameters (see Supplementary Method 2). In the case of constrained BB emissions (Supplementary Fig. 4), the retrieval error of AE was the leading contributor to the overall uncertainties (24% on average), followed by AOD retrieval errors (11%). We further repeated our constraining procedures over the Amazon region using AERONET data (other regions were neglected because of the poor AERONET coverage), for which the retrieval errors were assumed to be zero. A comparison between the results using AERONET and POLDER (Supplementary Fig. 9) suggested an agreement within their stated uncertainties, with slightly larger uncertainties found for the former. The larger uncertainties in the AERONET-based results resulted mainly from the uncertainty of regional values due to much smaller spatiotemporal data coverage.

### Estimation of AOD in the Africa outflow region

The AOD in the Atlantic outflow of the BBA from South Hemisphere Africa was modelled by multivariate linear regression from AeroCom model data. We assumed that the regional, fire-season average burden in the outflow region (*M*_*O*_) can be modelled as a linear process:

4 $${M}_{o}={\alpha E}_{s}{\tau }_{s}+\beta$$

where *E*_*s*_ and *τ*_*s*_ are the emission and lifetime of aerosols over the African source region, respectively, and *β* indicates the background aerosols (e.g., sea salt). For MEC over the southeast Atlantic region (*MEC*_*O*_), which is dominated by aged BBA, we used a simple linear relation with MEC over the source region (*MEC*_*s*_) to broadly represent the ageing process:

5 $${{MEC}}_{o}={\gamma {MEC}}_{s}+\theta$$

By combining Eqs. 4 and 5, the relation between AOD over Africa outflow region (*AOD*_*o*_) and aerosol from the source region can be written as follows:

6 $${{AOD}}_{o}=\left({\alpha E}_{s}{\tau }_{s}+\beta \right)\times \left({\gamma {MEC}}_{s}+\theta \right)=A\times {E}_{s}{\tau }_{s}{{MEC}}_{s}+B\times {E}_{s}{\tau }_{s}+C\times {{MEC}}_{s}+D$$

The coefficients (*A, B, C, D*) can be obtained from a multiple linear regression based on all model data. Supplementary Fig. 13 shows an evaluation of this simple model for the outflow AOD, suggesting reasonably good agreement. Then, we adopted the EC and MFC cases to correct AOD errors over the source region using Eq. (6). For the EC case, we only scaled emissions by modelled AOD errors relative to POLDER observations. In contrast, we changed the total emissions, lifetime, and MEC to the constrained values as estimated above in the MFC case.

### ECHAM-HAM simulations

The ECHAM-HAM model was run at T63 horizontal resolution, with 47 vertical layers with the M7 submodule for aerosol microphysics^54^. Emissions for major aerosol sources were taken from GFED for BB, the Community Emissions Data System for anthropogenic sources, the AeroCom phase II dataset for biogenic volatile organic compounds, and online calculations for dust and sea salt. Meteorology was nudged with ECMWF Reanalysis v5 (ERA5). The simulations covered the entire fire season for Southern Hemisphere Africa and started three months earlier for model spin-up. We compared three different cases within ECHAM-HAM (see Supplementary Table 6): 1) the default model configuration; 2) the EC case using default model configuration but with emissions over the Africa source regions scaled to match POLDER AOD observations; and 3) the MFC case with a modified model configuration based on the error attribution done for ECHAM-HAM. Different from the previous meta-model analysis where we could directly apply the constrained results (i.e., emission, lifetime, MEC), we had to modify model aspects for the MFC case in ECHAM-HAM simulations.

To correct the model’s MEC, we used a larger emitted BBA particle size than the default. This was based on the observed overestimation of AE that suggested that particles were too small by default. In practice, we increased the emitted particle size to 200 nm and slightly inflated the ambient size by 10% based on a series of experiments with various emitted and ambient sizes (see Supplementary Fig. 14). Although other solutions were also feasible (e.g., larger mode widths, increasing coagulation or condensation), the detailed way to modify the particle size did not matter here, as the most important aspect was to achieve a larger ambient particle (and larger MEC). Regarding lifetime, both precipitation and particle size could affect the deposition (and hence lifetime). In addition to the above correction on the particle size, we further scaled the wet deposition based on the observed precipitation error over the source region (−69%). We did not directly alter precipitation because it would result in changes in the entire hydrological cycle in the model. Again, the detailed way to rescale precipitation did not matter as long as the modelled lifetime was corrected towards the constrained value. Because BBA was the focus of this work, we only adopted the EC and MFC corrections to the BBA and the background aerosols were additionally simulated with a background run using the default configuration, as detailed in Supplementary Table 6.

## Supplementary information

Supplementary Information

## Data Availability

The AeroCom model data can be accessed from https://aerocom.met.no. POLDER observations can be downloaded from https://www.grasp-open.com/products/polder-data-release. GPCP data can be downloaded from https://climatedataguide.ucar.edu/climate-data/gpcp-daily-global-precipitation-climatology-project. BBA emissions from the four emission inventories can be downloaded from https://www.geo.vu.nl/~gwerf/GFED/GFED4 for GFED, https://apps.ecmwf.int/datasets/data/cams-gfas for GFAS, https://feer.gsfc.nasa.gov/data/emissions for FEER, https://portal.nccs.nasa.gov/datashare/iesa/aerosol/emissions/QFED/v2.5r1/0.1/QFED for QFED. Sources of independent observations can be found in the Supplementary Information. The ECHAM-HAM simulation data are deposited in Zenodo (10.5281/zenodo.7020733).

## Code availability

The ECHAM-HAM model source code can be accessed at Redmine: https://redmine.hammoz.ethz.ch. Data processing and analysis in this paper were conducted using the Community Intercomparison Suite (CIS, http://www.cistools.net).
