# Supplementary material for: Using modelled relationships and satellite observations to attribute modelled aerosol biases over biomass burning regions
Source: Nat Commun. 2022 Oct 7;13:5914. doi: 10.1038/s41467-022-33680-4 (PMC9547058; doi:10.1038/s41467-022-33680-4)
Supplement: Supplementary file 1 — Supplementary Information [file 41467_2022_33680_MOESM1_ESM.pdf]

Supplementary Information

Using modelled relationships and satellite observations to attribute modelled aerosol biases over biomass burning regions

Qirui Zhong[1*], Nick Schutgens[1], Guido R. van der Werf[1], Twan van Noije[2], Susanne E. Bauer[3,4], Kostas Tsigaridis[3,4], Tero Mielonen[5], Ramiro Checa-Garcia[6,7], David Neubauer[8], Zak Kipling[9], Alf Kirkevåg[10], Dirk J. L. Olivié[10], Harri Kokkola[5], Hitoshi Matsui[11], Paul Ginoux[12], Toshihiko Takemura[13], Philippe Le Sager[2], Samuel Rémy[14], Huisheng Bian[15,16], Mian Chin[16]

[1]Department of Earth Sciences, Vrije Universiteit Amsterdam, Amsterdam, The Netherlands.

[2]Royal Netherlands Meteorological Institute, De Bilt, The Netherlands.

[3]NASA Goddard Institute for Space Studies, New York City, NY, USA.

[4]Center for Climate Systems Research, Columbia University, New York City, NY, USA.

[5]Finnish Meteorological Institute, Kuopio, Finland.

[6]Laboratoire des Sciences du Climat et de l'Environnement, IPSL, Gif-sur-Yvette, France.

[7]Institute of Meteorology and Climatology, University of Natural Resources and Life Sciences, Vienna, Austria.

[8]Institute for Atmospheric and Climate Science, ETH Zurich, Zurich, Switzerland.

[9]European Centre for Medium-Range Weather Forecasts, Reading, UK.

[10]Norwegian Meteorological Institute, Oslo, Norway.

[11]Graduate School of Environmental Studies, Nagoya University, Nagoya, Japan.

[12]NOAA, Geophysical Fluid Dynamics Laboratory, Princeton, NJ, USA.

[13]Research Institute for Applied Mechanics, Kyushu University, Fukuoka, Japan.

[14]HYGEOS, Lille, France.

[15]University of Maryland, Baltimore County (UMBC), Baltimore, MD, USA.

[16]NASA Goddard Space Flight Center, Greenbelt, MD, USA.

* Correspondence to: Qirui Zhong (q.zhong@vu.nl).

Content

Supplementary Methods: 1–2

Supplementary Figures: 1–14

Supplementary Tables: 1–6

Supplementary References: 1–43

Supplementary Method 1 | Estimating regional AOD and AE

The regional AOD was estimated via a combination of models and raw POLDER data. For AeroCom models with high-resolution output (3-hourly or daily), we derived a linear regression between the model average of AOD at all times and grid boxes (regional AOD) and the model average of AOD collocated with POLDER observations (see Supplementary Fig. 10 for the example of Southern Hemisphere Africa). Then, the regional AOD observation was estimated by employing the average of raw POLDER data for the regression (see the dashed lines in Supplementary Fig. 10). The uncertainties in regional AOD are based on the confidence intervals of the predicted values. We found that the predictions had small uncertainties except for AOD in boreal regions (i.e., boreal North America, Eastern Siberia, as shown in Supplementary Fig. 4), where the uncertainties resulted from the very limited sampling coverage of the POLDER dataset (< 1%). Similarly, we also estimated the regional AE.

To test the robustness of this method, we removed one of the models to estimate regional AOD and AE and repeated it for all the models. The variations in the predicted regional AOD and AE resulting from the exclusion of individual models were very small (< 5%), suggesting that the method was robust and not dependent on the models chosen for the estimations.

Supplementary Method 2 | Uncertainties from individual factors

The following factors were considered regarding the overall uncertainties throughout the analysis:

- Retrieval uncertainties for AOD and AE, quantified as 10% and 0.22, respectively, based on a global validation against the AErosol RObotic NETwork (AERONET) dataset, given the very limited data availability of the AERONET dataset over BB regions[1];
- Uncertainty of the GPCP dataset, set as 9% according to Adler et al[2];
- Uncertainty of the regional average AOD and AE, quantified based on the regression confidence intervals, as shown in Supplementary Fig. 10;
- Uncertainties in predicting constrained lifetime and MEC, estimated using the regression confidence intervals (see Fig. 2 and Supplementary Fig. 1). Note that the confidence intervals based on the model data range do not necessarily reflect true uncertainty. We assumed that the linear regressions presented reasonable approximations of the real relationships. The individual uncertainty contributions (see Supplementary Fig. 4) showed that the overall uncertainties were dominated by satellite retrieval errors, suggesting that our assumption would not fundamentally alter the results.
- Uncertainties in the background emissions, see Supplementary Table 5.

Individual distributions were developed for each factor based on the above parameters. These individual uncertainties contribute to the overall uncertainties for constrained emissions, lifetime, MEC, and error attribution. The overall uncertainty was calculated via a Monte-Carlo approach by randomly drawing inputs with replacement from the distribution of each involved parameter 100,000 times (see Table 1 and Fig. 3). The uncertainty caused by a single factor was calculated by keeping the corresponding factor uncertain and eliminating the uncertainties of all the other factors in the calculations (see Supplementary Fig. 4). The uncertainties are presented as the ratio of interquartile to median values.

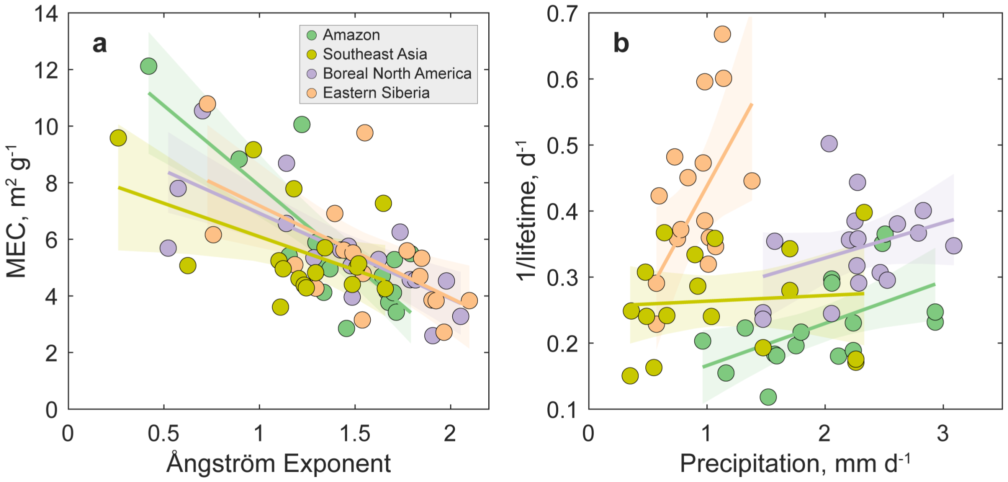

Supplementary Fig. 1 | Linear regressions for MEC over the Ångström Exponent (a) and lifetime over precipitation (b). The results are shown in the same format as Fig. 2 but for different regions as indicated by the colours. The solid lines indicate the linear regressions together with the 95% confidence intervals (shaded area). Note that the real lifetime regressions use both precipitation and Ångström Exponent as predictors.

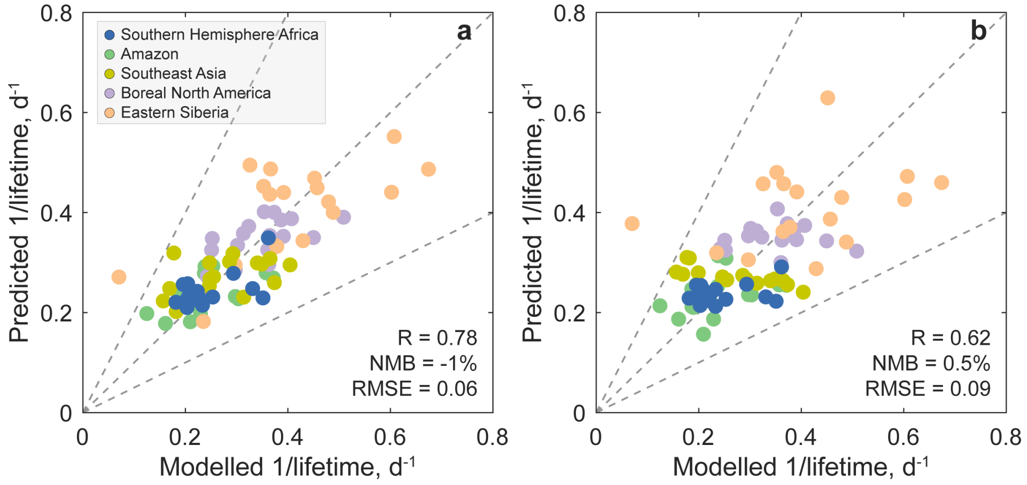

Supplementary Fig. 2 | Comparison between modelled and predicted 1/lifetime from our updated regressions that consider both precipitation and AE (a) and those based on precipitation only (b). The metrics for all the regions show the correlation coefficients (R), normalized mean bias (NMB), and root mean square error (RMSE).

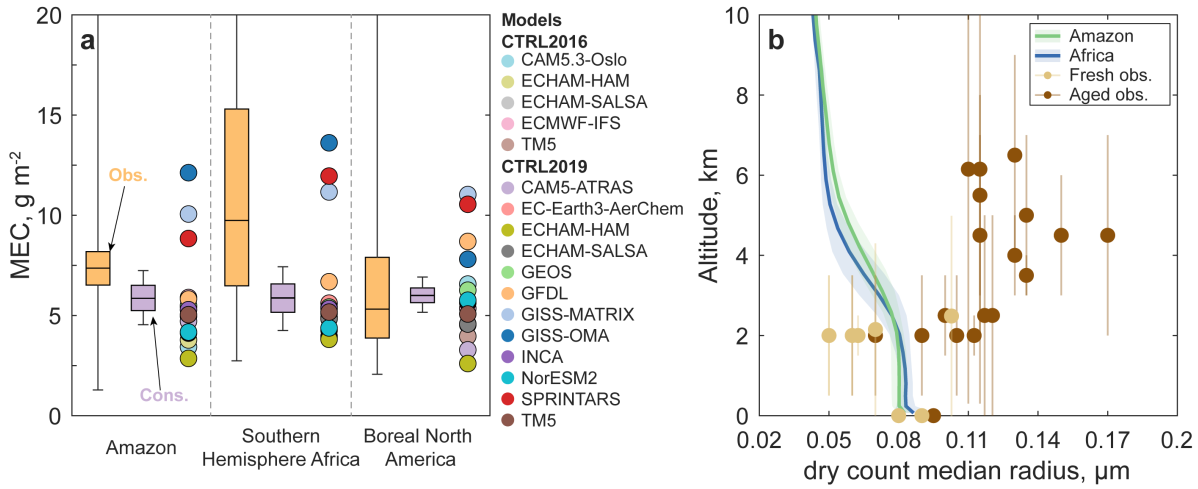

Supplementary Fig. 3 | Comparisons of MECs in AeroCom models (a) and dry count median radius in ECHAM-HAM (b) with flight campaign and field measurements. The observed MECs from flight campaigns (Obs.) and the constrained MECs in our analysis (Cons.) are shown as boxplots in Supplementary Fig. 3a. The data sources for flight campaigns can be found in Supplementary Tables 2-3. The modelled MECs over Africa are collected over the outflow region (see Fig. 1) in line with the flight campaign. The vertical bars of the observed radius in Supplementary Fig. 3b show the altitude ranges during the measurements, with the dots located at the median altitude. Vertical profiles of radius (mean ± standard deviation) over Amazon and Southern Hemisphere Africa in ECHAM-HAM are shown for comparison since most measurements are conducted for these two regions.

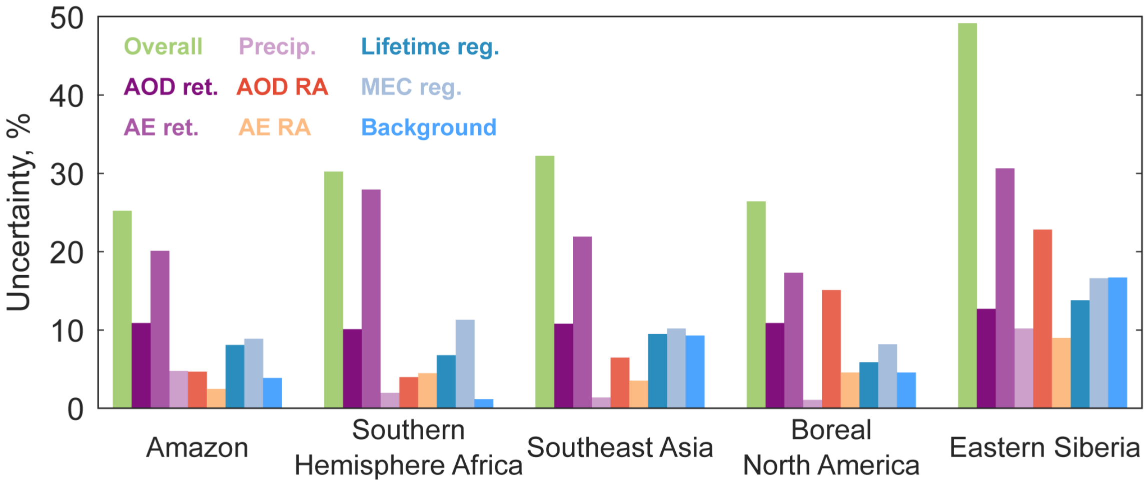

Supplementary Fig. 4 | Contribution of the eight factors to the overall uncertainty of constrained BBA emissions. The eight contributors are AOD retrieval errors (AOD ret.), AE retrieval errors (AE ret.), precipitation observation errors (Precip.), regional averages of AOD (AOD RA), regional averages of AE (AE RA), lifetime regression (Lifetime reg.), MEC regression (MEC reg.), and background emissions (Background). The uncertainty is calculated as the ratio of the interquartile to the median value from the Monte-Carlo estimates (see Methods).

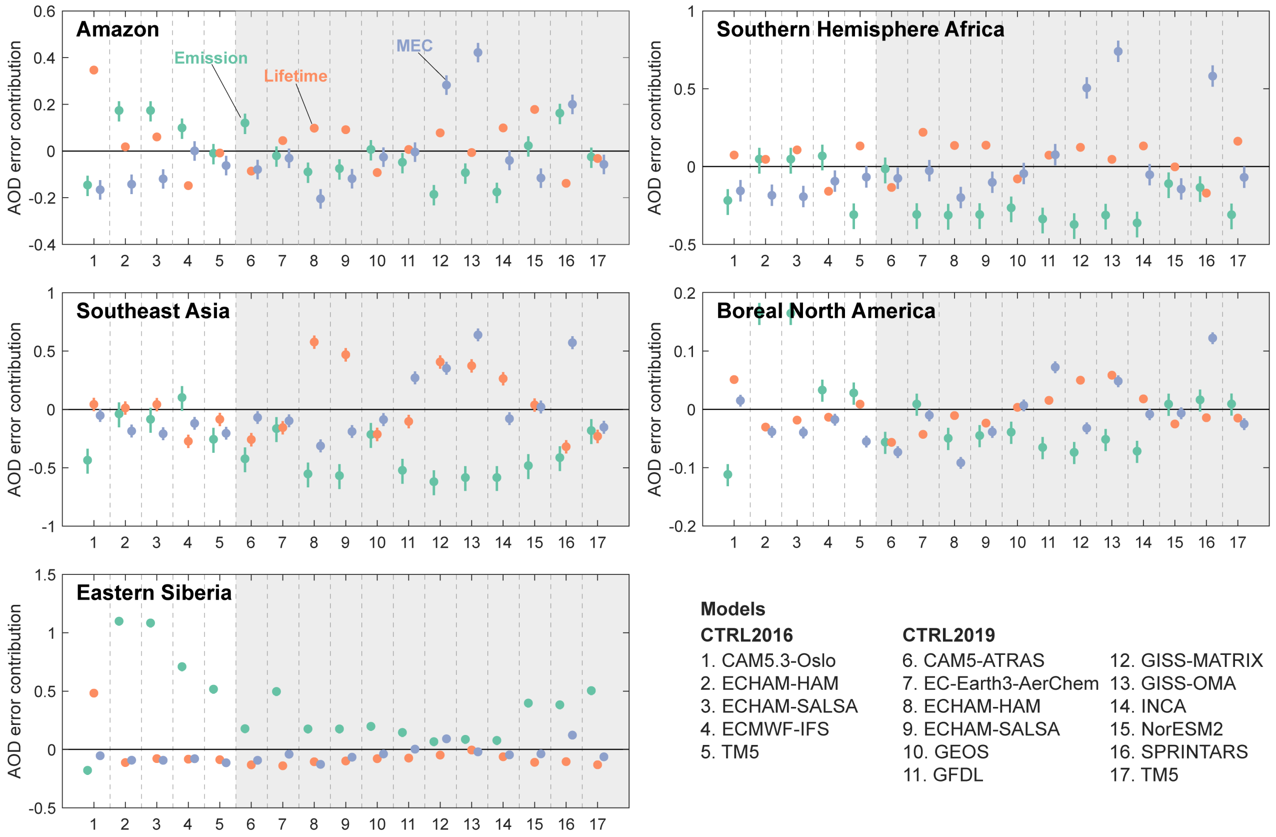

Supplementary Fig. 5 | The AOD errors attributed to emission, lifetime, and MEC over the five BB regions for the AeroCom models. The median contributions are shown as dots. Vertical bars denote the interquartile ranges of the error contribution of individual factors regarding the uncertainties of our error attributions.

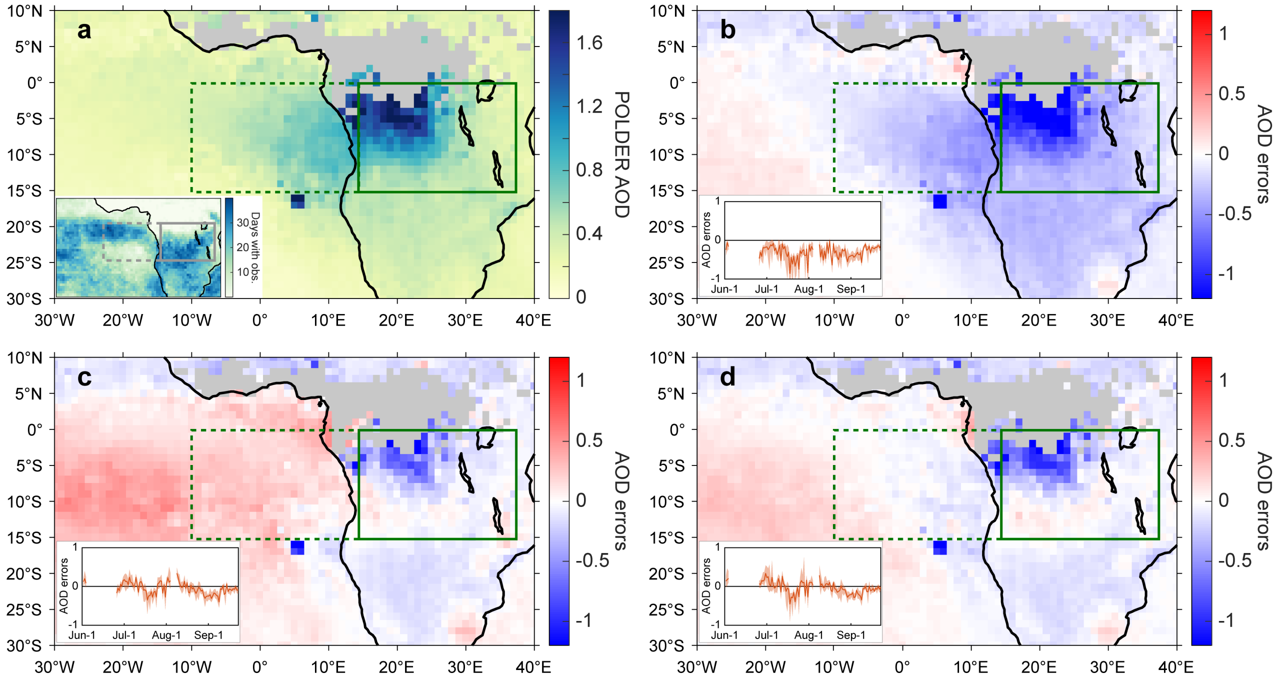

Supplementary Fig. 6 | Spatial distribution of POLDER observations of AOD (a) and the ECHAM-HAM modelled AOD errors in the default (b), EC (c), and MFC (d) cases. All the simulations are collocated and compared with POLDER AOD data (Supplementary Fig. 6a) that are sparsely distributed (https://www.grasp-open.com)[3]. The number of days with available POLDER AOD observation for each grid box is shown in the embedded diagram in Supplementary Fig. 6a. The fire-season average AOD values based on available samplings are shown in the maps. The boxes with solid edges show the African source area, and the boxes with dashed edges indicate the focused outflow region. The embedded diagrams in Supplementary Fig. 6b-d show the daily series of AOD errors for the source region as median (solid lines) and interquartile (shaded areas).

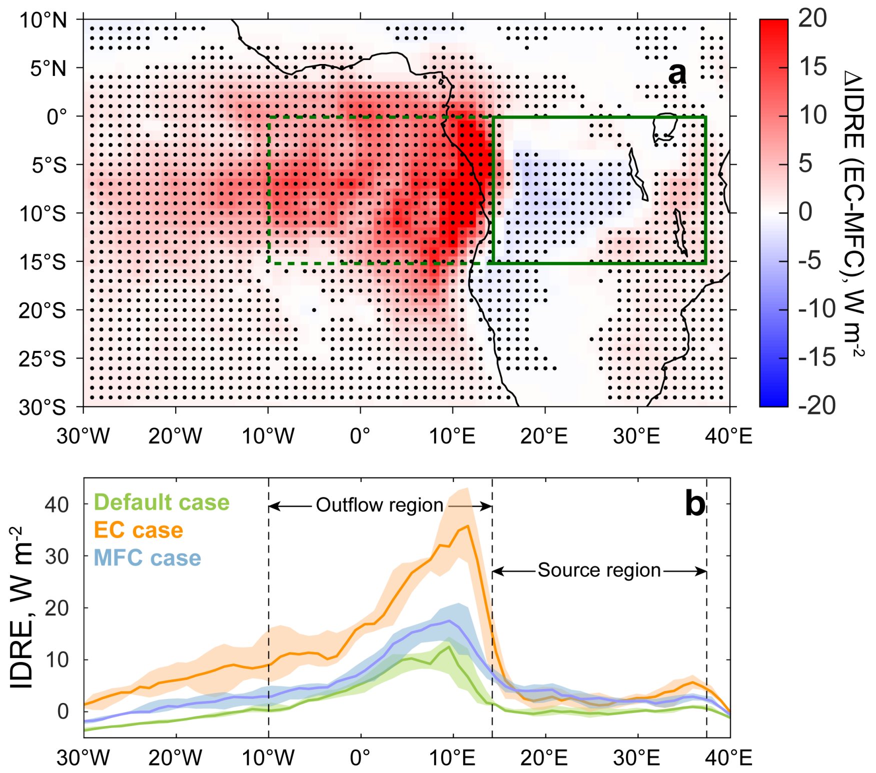

Supplementary Fig. 7 | Modelled difference of the instantaneous direct radiative effect (IDRE) between the EC and MFC cases over Africa (a) and the longitudinal IDRE (b) in ECHAM-HAM. The IDRE at the top of the atmosphere is calculated under all-sky conditions. The dots in the map indicate the grid cells with significant differences between the two cases ($p < 0.05$) based on daily series. The longitudinal IDREs in Supplementary Fig. 7b are shown as medians (solid lines) and interquartile ranges (shaded areas).

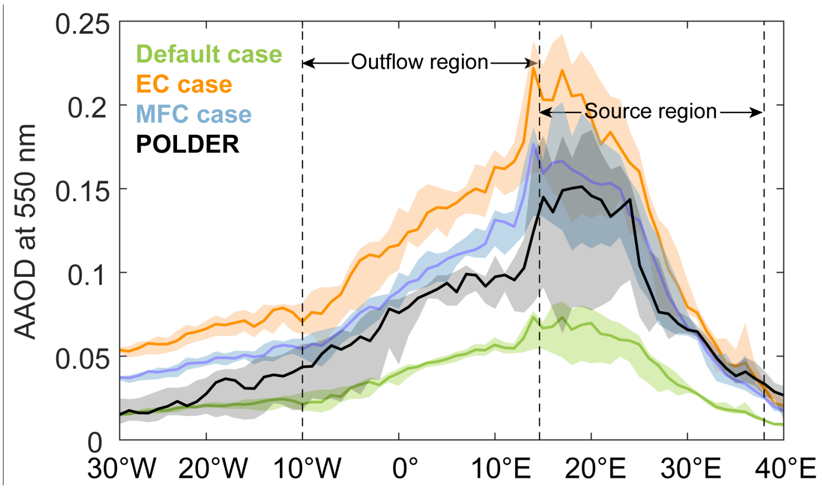

Supplementary Fig. 8 | Longitudinal average of the ECHAM-HAM modelled absorbing aerosol optical depth (AAOD) at 550 nm in the three cases and from POLDER observations over the African source region and the outflow region. All the modelled data are collocated with POLDER. The ranges of the source and outflow regions are shown in Supplementary Fig. 6. The data are shown for the whole fire season as the median (solid lines) and interquartile range (shaded areas).

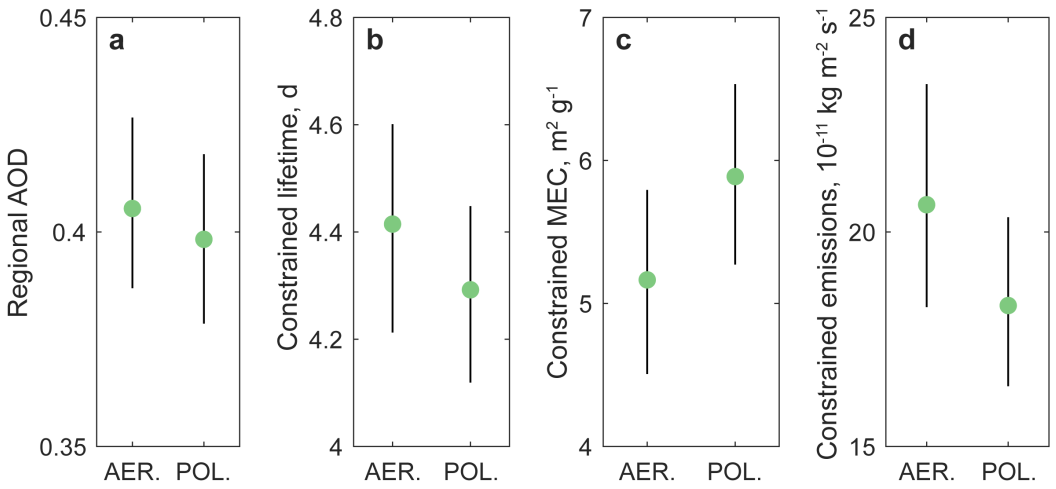

Supplementary Fig. 9 | Comparisons of regional AOD (a), constrained lifetime (b), constrained MEC (c), and constrained total emissions (b) by using POLDER (POL.) or AERONET (AER.) dataset in the Amazon. Error bars show the interquartile ranges. The uncertainties of AERONET-based results are dominated by the uncertainties in the regional AOD and AE due to limited sampling coverage, while the uncertainties of POLDER-based results are mainly contributed by the satellite retrieval errors (see Supplementary Fig. 4).

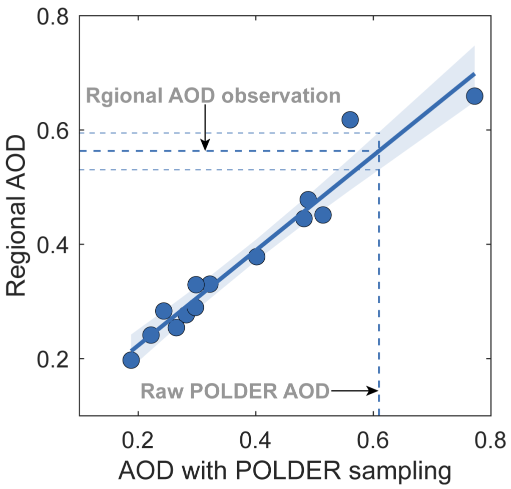

Supplementary Fig. 10 | Linear regressions of the modelled average of regional AOD over AOD collocated with POLDER sampling in Southern Hemisphere Africa. Each dot indicates data from one model. The solid line indicates the linear regression together with the 95% confidence interval (shaded area). The average of the raw POLDER data is shown as the vertical dashed line, and the corresponding predicted regional AOD and 95% confidence interval are shown as horizontal dashed lines. The regional AOD is the estimate of the average AOD for all times and grid-boxes in a fire season and region. This estimation is conducted for both AOD and AE over all the five BB regions during the fire seasons.

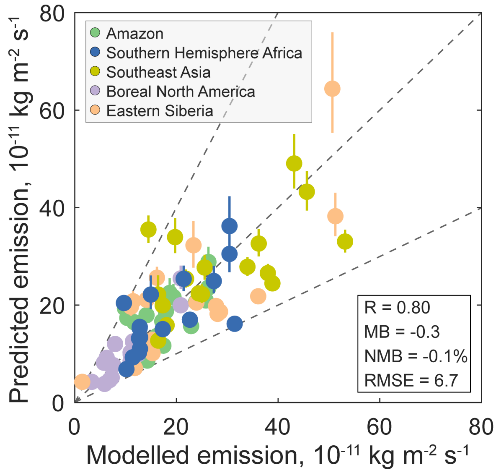

Supplementary Fig. 11 | Comparison of predicted total aerosol emissions with original model emissions. The predicted emission for each model is estimated using its modelled AOD and the predicted lifetime and MEC from the regressions based on the rest AeroCom models. Error bars denote the interquartile ranges from our estimations. The dashed lines indicate the 1:1, 1:2, and 2:1 ranges. Metrics for the regression validation include the Pearson correlation coefficient (R), mean bias (MB), normalized mean bias (NMB), and root mean square error (RMSE).

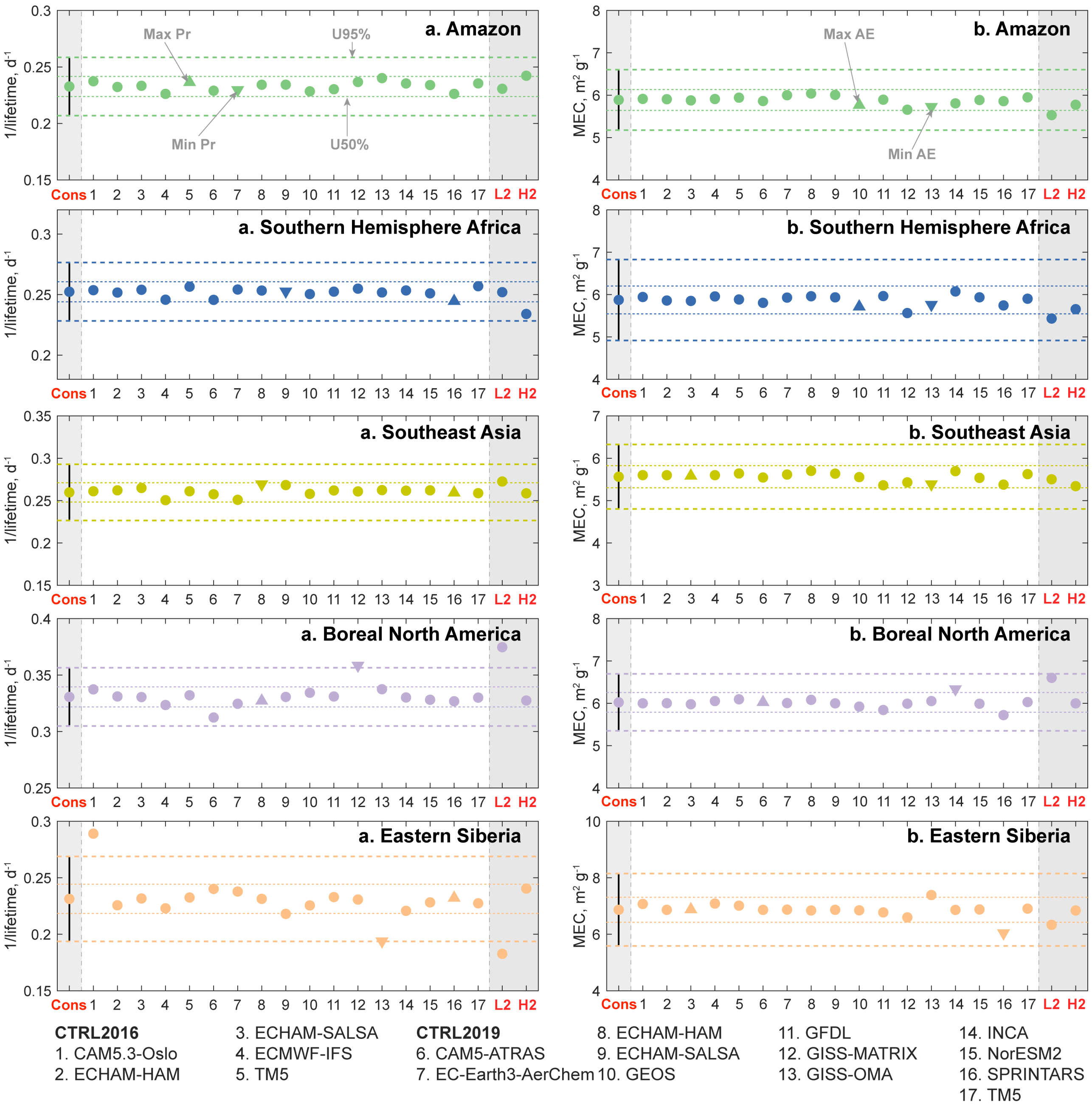

Supplementary Fig. 12 | Comparisons of the constrained 1/lifetime (a) and MEC (b) predicted using either all models (Cons) or those with one or two models excluded from the ensemble (1-17, L2, H2). The predictions using all models (Cons) are used in the main text. Vertical bars and horizontal lines denote the 95% (dashed lines, U95%) and 50% (dotted lines, U50%) uncertainty intervals for the predictions. Dots at 1-17 show the predictions when the corresponding model is removed from the calculations. The model numbers are shown at the bottom. The upwards and downwards triangles indicate the models with the maximum and minimum precipitation (AE) in Supplementary Fig. 12a (Supplementary Fig. 12b), respectively. L2 and H2 in Supplementary Fig. 12a (Supplementary Fig. 12b) indicate the predicted lifetime (MEC) when leaving out the two models with the lowest or highest precipitation (AE), respectively.

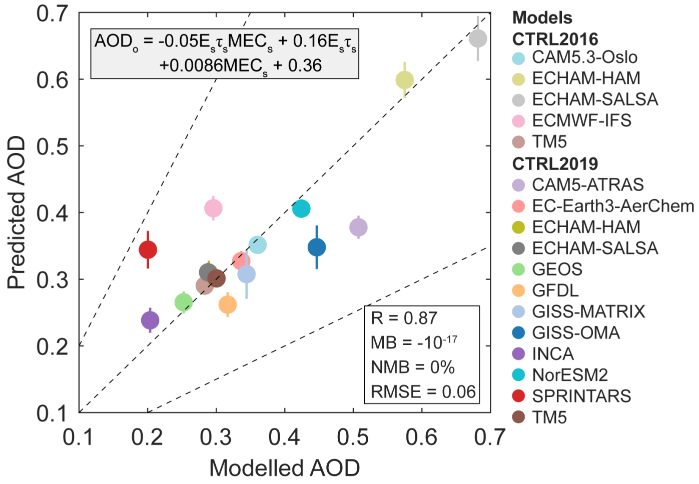

Supplementary Fig. 13 | Validation of the regression for the AOD over the Africa outflow region (the Southeast Atlantic, see Supplementary Fig. 6). The outflow AOD (AOD_O) is regressed against the emission (E_S), lifetime (τ_S), and MEC (MEC_S) over the source region. The vertical bars show the 50% confidence intervals from the regression. Metrics for the regression validation include the Pearson correlation coefficient (R), mean bias (MB), normalized mean bias (NMB), and root mean square error (RMSE). The equation shows the regression coefficients for Eq. 6.

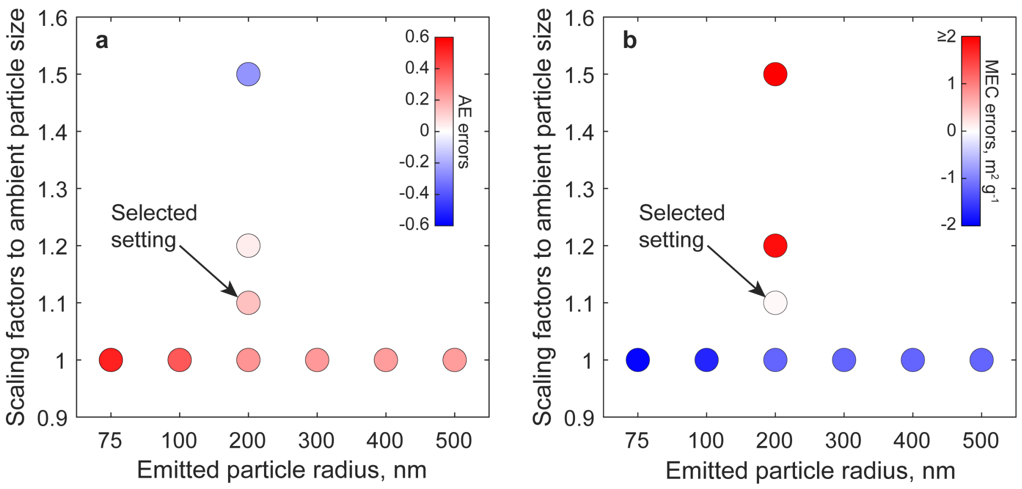

Supplementary Fig. 14 | ECHAM-HAM modelled errors over Southern Hemisphere Africa for AEs (a) and MECs (b) with different emitted particle radii for BBA (from 75 nm to 500 nm) and different scaling factors to the ambient particle size. The emitted particle radius indicates the number median radius for the log-normal size distribution. The errors for AE (Supplementary Fig. 14a) and MEC (Supplementary Fig. 14b) are indicated by the colour of the dots. The combination of a 200 nm emitted particle radius and a scaling factor of 1.1 was selected to present the MFC in ECHAM-HAM given the agreement with our constrained MEC.

Supplementary Table 1 | Details of the 17 AeroCom models used in this study. All models present simulations for 2010. Additional requirements of using CMIP6 (Coupled Model Intercomparison Project, Phase 6) emissions are proposed in the CTRL2019 experiment.

Experiment	Models	Grid[a]	Output frequency	Meteorology	BB emission[b]	OA/OC	Refs	Normalized mean bias of AOD, %[c]				
								Amazon	Southern Hemisphere Africa	South-east Asia	Boreal North America	Eastern Siberia
CTRL2016	CAM5.3-Oslo	192×288×30	3-hour	ERA-Interim	CMIP5	2.6	4	-43	-58	-53	-79	-84
	ECHAM-HAM	96×192×47	3-hour	ERA-Interim	3.4×GFAS	1.4	5	-12	-19	-25	62	324
	ECHAM-SALSA	96×192×47	3-hour	ERA-Interim	AeroCom-II	1.4	6	7	-17	-26	108	716
	ECMWF-IFS	256×512×60	3-hour	ECMWF	3.4×GFAS	1.8	7	-33	-35	-30	4	61
	TM5	90×120×34	3-hour	ERA-Interim	GFED	1.6	8	-28	-51	-48	-59	-16
CTRL2019	CAM5-ATRAS	96×144×30	Daily	MERRA2	CMIP6	1.4	9	-23	-44	-62	-86	-61
	EC-Earth3-AerChem	90×120×34	Monthly	ECMWF	CMIP6	1.6	10					
	ECHAM-HAM	96×192×47	Daily	ERA-Interim	CMIP6	1.4	5	-60	-65	-58	-85	-64
	ECHAM-SALSA	96×192×47	Daily	ERA-Interim	CMIP6	1.4	6	-38	-53	-54	-73	-29
	GEOS	181×360×72	Daily	MERRA2	CMIP6	1.8	11	-36	-61	-47	-60	-35
	GFDL	180×288×33	Monthly	NCEP/NCAR	CMIP6	1.6	12					
	GISS-MATRIX	90×144×40	Daily	NCEP/NCAR	CMIP6	1.4	13	23	-14	-31	-26	37
	GISS-OMA	90×144×40	Daily	NCEP/NCAR	CMIP6	1.4	14	77	23	-7	-42	2
	INCA	143×144×79	Monthly	ERA-Interim	CMIP6	1.4	15					
	NorESM2	192×288×32	Daily	ERA-Interim	CMIP6	2.6	16	-18	-47	-49	-49	-1
	SPRINTARS	320×640×40	Daily	ERA5	CMIP6	2.6	17	28	-2	-44	-1	18
	TM5	90×120×34	Daily	ERA-Interim	CMIP6	1.6	8, 10	-26	-48	-43	-59	-40

a. Grid structure is shown as latitude × longitude × vertical layer.
b. The BB emission sources provide OC emissions and models need to convert to the OA emissions based on their assumptions on OA/OC ratios, which lead to different emission inputs even using same inventories.
c. Data are the regional, fire-season averages, with models collocated and compared with POLDER dataset. Biases for models with monthly frequency are not shown given the sampling issues.

Supplementary Table 2 | Flight campaigns used in this study for MEC validations.

Campaigns	Data source	Time	Regions	Wavelength	Reference
SAMBBA	South AMerican Biomass Burning Analysis	2012/09-2012/10	South America	550 nm	18
ORACLES	ObseRvations of Aerosols above CLouds and their intEractionS	2016/08-2016/10<br>2017/08-2017/09<br>2018/09-2018/10	Southeast Atlantic (outflow of Southern Hemisphere Africa)	550/530 nm[a]	19
ARCTAS	Arctic Research of the Composition of the Troposphere from Aircraft and Satellites	2008/06-2008/07	Boreal North America	550/532 nm[b]	20

a. Light scattering and absorption are collected at 550 and 530 nm, respectively.
b. Light scattering and absorption are collected at 550 and 532 nm, respectively.

Supplementary Table 3 | Number median radius for BBA particles collected from field measurements.

Regions	Biome	Instrument[a]	Altitude, km	Category	Radius, μm	Reference
Africa	Savanna	PCASP	0.3~12	Aged	0.11	21
Brazil	Forest	PCASP	0.3~12	Aged	0.115	21
Greece	Forest	PCASP	1.5~3.5	Aged	0.1	22
Ascension	Grass	PCASP	0~5	Aged	0.117	23
Atlantic	Grass	PCASP	0~5	Aged	0.120378	23
Temperate	Prescribed	DMPS		Aged	0.095	24
South Africa	Grass	PCASP	1.5~2.5	Aged	0.1125	25
Europe	Forest	PCASP	3~4	Aged (10-13 day)	0.135	26
Europe	Forest	PCASP	3~8	Aged (4-6 day)	0.115	26
Europe	Forest	PCASP	4~9	Aged (6-9 day)	0.13	26
Europe	Forest	PCASP	3~7	Aged (6-9 day)	0.135	26
Europe	Forest	PCASP	3~5	Aged (6-9 day)	0.13	26
Europe	Forest	PCASP	3~6	Aged (7-10 day)	0.15	26
Brazil	Forest	PCASP	0.5~3.5	Aged (1 day)	0.06	27
Brazil	Forest	PCASP	0.5~3.5	Aged (1 day)	0.07	27
Brazil	Forest	PCASP	0.5~3.5	Aged (2 day)	0.09	27
Brazil	Forest	PCASP	0.5~3.5	Aged (2 day)	0.105	27
North Canada	Forest	PCASP	2~7	Well-aged	0.17	28
North Canada	Forest	PCASP	2~7	Well-aged	0.115	28
Temperate	Prescribed	OPC		Fresh	0.09	29
Amazon	Forest	SMPS	<4.3	Fresh	0.07	30
Temperate	Prescribed	DMPS		Fresh	0.08	24
South Africa	Grass	PCASP	1.5~2.5	Fresh	0.0625	25
Temperate	Prescribed	SEM		Fresh	0.09	31
Brazil	Grass	DMPS	0.5~3.5	Fresh (< 4 min)	0.05	27
Brazil	Cerrado	DMPS	0.5~3.5	Fresh (< 4 min)	0.05	27
Brazil	Forest	DMPS	0.5~3.5	Fresh (< 4 min)	0.06	27

a. The instruments/methods used to obtain the particle size distribution: PCASP (PMS Passive Cavity Aerosol Spectrometer Probe), DMPS (Differential Mobility Particle Sizer), OPC (Optical Particle Counter), SMPS (Scanning Mobility Particle Sizer), SEM (Scanning Electron Microscopy).

Supplementary Table 4 | Comparison of the IDRE at the top of the atmosphere under all-sky conditions between this study and previous literature.

Source	Time	Method	IDRE, W/m^2
Keli & Haywood, 2003[32]	07-09/2000	Estimated based on SAFARI 2000 campaign	11.5
Chand et al., 2009[33]	07-10/2006-2007	Estimated based on CALIPSO and Terra observation	2.4
Wilcox, 2012[34]	07-09/2005-2006	Estimated based on satellite observation	9.2 ± 6.6
Meyer et al., 2013[35]	08-09/2006-2011	Estimated based on MODIS and CALIOP observation	14.9 ± 17.0
Marquardt Collow et al., 2020[36]	07-10/2016-2017	Estimated based on MERRA-2 and field measurements	-20
Doherty et al., 2022[37]	09/2016; 08/2017; 08/2018	Predicted based on ORACLES observation	2.1~16.1
This study	06-09/2010	Default case (ECHAM-HAM with GFED4.1s)<br>EC case<br>MFC case	5.8 ± 5.0<br>20.5 ± 11.5<br>9.5 ± 5.1

Supplementary Table 5 | Background aerosol emissions over the five BB regions during BB seasons. The median values together with interquartile ranges (in brackets) are shown when multiple data sources are available.

Background	Emissions (10^{-11} kg m^{-2} s^{-1})					Data source	Reference
	Amazon	Southern Hemisphere Africa	Southeast Asia	Boreal North America	Eastern Siberia		
Anthropogenic[a]	0.065	0.46	0.99	0.0076	0.00072	CEDS	38
Biogenic[b]	1.24 (1.20, 1.46)	0.37 (0.27, 0.54)	0.70 (0.63, 0.74)	0.30 (0.27, 0.39)	0.37 (0.27, 0.54)	AeroCom-II MEGANv2 MEGAN-MACC CAMS-GLOB-BIO	39-42
Dust	0.0013 (0.0001, 0.16)	0	0	0	0	17 AeroCom models	See Supplementary Table 1
Sea salt[c]	0.011 (0, 0.25)	0.008 (0, 0.05)	3.32 (1.91, 7.64)	0.04 (0, 0.16)	0.46 (0.13, 1.1)	17 AeroCom models	See Supplementary Table 1
Total	1.32	0.84	5.01	0.35	0.83		

a. The uncertainties of anthropogenic emissions are set as 80% according to Huang et al (2015)[43]. It considers the same aerosol components as BBA (OA, BC, and SO_2).

b. The biogenic emissions are calculated as 15% of the emitted mass of terpenes to indicate the biogenic SOA[39].

c. The sea salt emissions over Amazon, Africa, and Siberia result from the grid boxes that cover both land and ocean areas in original models given the rather coarse resolutions (see Supplementary Table 1).

Supplementary Table 6 | Major parameterizations for the ECHAM-HAM simulations.

Cases	BBA Emission	Emitted size of BBA	Ambient size[a]	Precipitation[b]
Default	GFED4.1s	75 nm	Default	Default
Background	No BBA emissions	NA	Default	Default
EC	2.9×GFED4.1s	75 nm	Default	Default
MFC	2.3×GFED4.1s	200 nm	Scaled by 1.1	Scaled by 3.3

a. The ambient particle size is only modified for calculations of optical properties, wet, and dry deposition.
b. The scaling factor of precipitation is directly applied to wet removal.

References

1. Schutgens, N. et al. AEROCOM and AEROSAT AAOD and SSA study – Part 1: Evaluation and intercomparison of satellite measurements. *Atmos. Chem. Phys.* **21,** 6895-6917 (2021).
2. Adler, R. F., Gu, G. & Huffman, G. J. Estimating climatological bias errors for the Global Precipitation Climatology Project (GPCP). *J. Appl. Meteorol. Climatol*. **51,** 84-99 (2012).
3. Dubovik, O. et al. Statistically optimized inversion algorithm for enhanced retrieval of aerosol properties from spectral multi-angle polarimetric satellite observations. *Atmos. Meas. Tech.* **4,** 975-1018 (2011).
4. Kirkevåg, A. et al. A production-tagged aerosol module for Earth system models, OsloAero5.3 - extensions and updates for CAM5. 3-Oslo. *Geosci. Model Dev.* **11,** 3945-3982 (2018).
5. Tegen, I. et al. The global aerosol–climate model ECHAM6.3-HAM2.3-Part 1: Aerosol evaluation. *Geosci. Model Dev.* **12,** 1643-1677 (2019).
6. Kokkola, H. et al. SALSA2.0: The sectional aerosol module of the aerosol-chemistry-climate model ECHAM6.3.0-HAM2.3-MOZ1.0. *Geosci. Model Dev.* **11,** 3833-3863 (2018).
7. Rémy, S. et al. Description and evaluation of the tropospheric aerosol scheme in the European Centre for Medium-Range Weather Forecasts (ECMWF) Integrated Forecasting System (IFS-AER, cycle 45R1). *Geosci. Model Dev.* **12,** 4627-4659 (2019).
8. van Noije, T. P. C. et al. Simulation of tropospheric chemistry and aerosols with the climate model EC-Earth. *Geosci. Model Dev.* **7,** 2435-2475 (2014).
9. Matsui, H. Development of a global aerosol model using a two-dimensional sectional method: 1. Model design. *J. Adv. Model. Earth Syst.* **9,** 1921-1947 (2017).
10. van Noije, T. et al. EC-Earth3-AerChem: a global climate model with interactive aerosols and atmospheric chemistry participating in CMIP6. *Geosci. Model Dev.* **14,** 5637-5668 (2021).
11. Colarco, P., da Silva, A., Chin, M., & Diehl, T. Online simulations of global aerosol distributions in the NASA GEOS-4 model and comparisons to satellite and ground-based aerosol optical depth. *J. Geophys. Res. Atmos.* **115,** D14207 (2010).
12. Zhao, M. et al. The GFDL Global Atmosphere and Land Model AM4.0/LM4.0: 2. Model Description, Sensitivity Studies, and Tuning Strategies, *J. Adv. Model. Earth Sys.* **10,** 735-769 (2018).
13. Bauer, S. E. et al. MATRIX (Multiconfiguration Aerosol TRacker of mIXing state): an aerosol microphysical module for global atmospheric models. *Atmos. Chem. Phys.* **8,** 6003-6035 (2008).
14. Bauer, S. E. et al. Historical (1850–2014) Aerosol Evolution and Role on Climate Forcing Using the GISS ModelE2.1 Contribution to CMIP6. *J. Adv. Model. Earth Syst.* **8,** e2019MS001978 (2020).
15. Schulz, M., Cozic, A. & Szopa, S. LMDzT-INCA dust forecast model developments and associated validation efforts. *IOP Conference Series: Earth and Environmental Science* **7,** 12014 (2009).
16. Seland, Ø. et al. Overview of the Norwegian Earth System Model (NorESM2) and key climate response of CMIP6 DECK, historical, and scenario simulations. *Geosci. Model Dev.* **13,** 6165-6200, (2020).
17. Takemura, T., Nozawa, T., Emori, S., Nakajima, T. Y. & Nakajima, T. Simulation of climate response to aerosol direct and indirect effects with aerosol transport-radiation model. *J. Geophys. Res. Atmos.* **110,** D02202 (2005).
18. Facility for Airborne Atmospheric Measurements; Natural Environment Research Council; Met Office; Coe, H. (2014): SAMBBA: in-situ airborne observations by the FAAM BAE-146 aircraft. NCAS British Atmospheric. http://catalogue.ceda.ac.uk/uuid/2ff89840a89840868acff801f8859451 (accessed 2020-10).
19. Redemann, J. et al. An overview of the ORACLES (ObseRvations of Aerosols above CLouds and their intEractionS) project: aerosol–cloud–radiation interactions in the southeast Atlantic basin. *Atmos. Chem. Phys.*, **21,** 1507-1563 (2021).
20. Jacob, D. J. et al. The Arctic Research of the Composition of the Troposphere from Aircraft and Satellites (ARCTAS) mission: design, execution, and first results. *Atmos. Chem. Phys.* **10,** 5191-5212 (2010).
21. Anderson, B. E. et al. Aerosols from biomass burning over the tropical South Atlantic region: Distributions and impacts. *J. Geophys. Res. Atmos.* **101,** 24117-24137 (1996).

22. Formenti, P. et al. STAAARTE-MED 1998 summer airborne measurements over the Aegean Sea, 2, Aerosol scattering and absorption, and radiative calculations, *J. Geophys. Res.* **107,** 4451 (2002).

23. Haywood, J. M. et al. The mean physical and optical properties of regional haze dominated by biomass burning aerosol measured from the C-130 aircraft during SAFARI 2000, *J. Geophys. Res.* **108,** 8473 (2003).

24. Hobbs, P. V. et al. Particle and trace-gas measurements in smoke from prescribed burns of forest products in the Pacific Northwest, in: *Biomass Burning and Global Change*. **1,** 697-715, MIT Press, New York, 1006 (1996).

25. Le Canut, P. et al. Airborne studies of emissions from savanna fires in southern Africa: 1. Aerosol emissions measured with a laser optical particle counter. *J. Geophys. Res. Atmos.* **101,** 23615-23630 (1996).

26. Petzold, A. et al. Perturbation of the European free troposphere aerosol by North American forest fire plumes during the ICARTT-ITOP experiment in summer 2004. *Atmos. Chem. Phys.* **7,** 5105-5127 (2007).

27. Reid, J. S. & Hobbs, P. V. Physical and optical properties of young smoke from individual biomass fires in Brazil. *J. Geophys. Res. Atmos.* **103,** 32013-32030 (1998).

28. Fiebig, M., Stohl, A., Wendisch, M., Eckhardt, S. & Petzold, A. Dependence of solar radiative forcing of forest fire aerosol on ageing and state of mixture. *Atmos. Chem. Phys.* **3,** 881-891 (2003).

29. Einfeld, W., Ward, D. E. & Hardy, C. Effects of fire behavior on prescribed fire smoke characteristics: A case study, in: *Global biomass burning: Atmospheric, climatic, and biospheric implications.* Cambridge, MA: MIT Press. 412-419 (1991).

30. Guyon, P. Airborne measurements of trace gas and aerosol particle emissions from biomass burning in Amazonia. *Atmos. Chem. Phys.* **5,** 2989-3002 (2005).

31. Martins, J. V. et al. Particle size distributions, elemental compositions, carbon measurements, and optical properties of smoke from biomass burning in the Pacific Northwest of the United States, in: *Global Biomass Burning and Global Change*. 716-732, MIT Press, Cambridge, Mass (1996).

32. Keil, A & Haywood, J. M. Solar radiative forcing by biomass burning aerosol particles during SAFARI 2000: A case study based on measured aerosol and cloud properties. *J. Geophys. Res. Atmos.* **108,** D13 (2003).

33. Chand, D., Wood, R., Anderson, T. L., Satheesh, S. K. & Charlson, R. J. Satellite-derived direct radiative effect of aerosols dependent on cloud cover. *Nat. Geosci.* **2,** 181-184 (2009).

34. Wilcox, E. M. Direct and semi-direct radiative forcing of smoke aerosols over clouds. *Atmos. Chem. Phys.* **12,** 139-149 (2012).

35. Meyer, K., Platnick, S., Oreopoulos, L. & Lee, D. Estimating the direct radiative effect of absorbing aerosols overlying marine boundary layer clouds in the southeast Atlantic using MODIS and CALIOP. *J. Geophys. Res. Atmos.* **118,** 4801-4815 (2013).

36. Marquardt Collow, A. B., Miller, M. A., Trabachino, L. C., Jensen, M. P. & Wang, M. Radiative heating rate profiles over the southeast Atlantic Ocean during the 2016 and 2017 biomass burning seasons. *Atmos. Chem. Phys.* **20,** 10073-10090 (2020).

37. Doherty, S. J. et al. Modeled and observed properties related to the direct aerosol radiative effect of biomass burning aerosol over the southeastern Atlantic. *Atmos. Chem. Phys.* 22, 1-46 (2022).

38. McDuffie, E. E. et al. A global anthropogenic emission inventory of atmospheric pollutants from sector- and fuel-specific sources (1970–2017): an application of the Community Emissions Data System (CEDS). *Earth Syst. Sci. Data* **12,** 3413-3442 (2020).

39. Dentener, F. et al. Emissions of primary aerosol and precursor gases in the years 2000 and 1750 prescribed data-sets for AeroCom. *Atmos. Chem. Phys.* **6,** 4321-4344 (2006).

40. Bauwens, M. et al. Recent past (1979-2014) and future (2070-2099) isoprene fluxes over Europe simulated with the MEGAN–MOHYCAN model. *Biogeosciences* **15,** 3673-3690 (2018).

41. Guenther, A. B. et al. The Model of Emissions of Gases and Aerosols from Nature version 2.1 (MEGAN2.1): an extended and updated framework for modeling biogenic emissions. *Geosci. Model Dev.* **5,** 1471-1492 (2012).

42. Sindelarova, K., Markova, J., Simpson, D., Huszar, P., Karlicky, J., Darras, S., Granier, C.: Copernicus Atmosphere Monitoring Service Global Biogenic VOC emissions version 3.1 (CAMS-GLOB-BIOv3.1), ECCAD, https://doi.org/10.24380/cv4p-5f79 (2021).

43. Huang, Y. et al. Global organic carbon emissions from primary sources from 1960 to 2009. *Atmos. Environ.* **122,** 505-512 (2015).
